# Collaborating with and enabling diverse communities to address health inequities: The experiences of a community engagement and outreach team

**DOI:** 10.1017/cts.2025.7

**Published:** 2025-01-22

**Authors:** Reimund Serafica, Lorraine S. Evangelista, Tony Ward, Jeffery Peterson, Joseph Guerrero Lopez, Julie Lucero, Esther Erdei, Kathryn L. Braun, Andrea Bersamin, Jenifer Thomas, J.D. Wulfhorst, Cheryl Jorcyk, Rebecca Palacios, Judith Owens-Manley, Elizabeth Fore, Ann Bertagnolli, Chelsea Bellon, Francisco S. Sy

**Affiliations:** 1 School of Nursing and Mountain West CTR-IN, University of Nevada, Las Vegas, NV, USA; 2 Mountain West CTR-IN, University of Nevada, Las Vegas, NV, USA; 3 School of Public and Community Health Sciences, University of Montana, Missoula, MT, USA; 4 Department of Environmental and Occupational Health, School of Public Health, University of Nevada, NV, Las Vegas, USA; 5 Department of Community Health Sciences, College of Liberal Arts, University of Nevada, Reno, NV, USA; 6 University of New Mexico Health Sciences Center, Native Environmental Health Equity Community Engagement and Dissemination Core, Albuquerque, NM, USA; 7 Office of Public Health Studies, University of Hawaii at Manoa, Honolulu, HI, USA; 8 Department of Biology and Wildlife, Center for Alaska Native Health Research (CANHR), University of Alaska, Fairbanks, AK, USA; 9 Fay W Whitney School of Nursing, University of Wyoming, Laramie, WY, USA; 10 Department of Natural Resources and Society, University of Idaho, Moscow, ID, USA; 11 Department of Biological Sciences, Boise State University, ID, Boise, USA; 12 Department of Public Health Sciences, New Mexico State University, Las Cruces, NM, USA; 13 School of Social Work, University of AlaskaAnchorage, AK, USA; 14 Idaho State University, Pocatello, ID, USA; 15 Montana State University, Bozeman, MT, USA; 16 Western Montana Area Health Education Center, University of Montana, Missoula, MT, USA

**Keywords:** Community-based research, community engagement and outreach, academic-community partnership, health inequities, underserved communities

## Abstract

The Mountain West Clinical and Translational Infrastructure Network Community Engagement and Outreach (CEO) Core has fostered academic-community engagement since 2018. States historically receiving lower levels of NIH funding are characterized by significantly higher proportions of rural and remote populations, as well as uniquely elevated percentages of Native American/Alaska Native and Native Hawaiian/Pacific Islander populations compared to most other states. This case study highlights the Core’s efforts in advancing community-engaged research. Key initiatives included forming a CEO Core Steering Committee to recruit interdisciplinary investigators, establishing regional community advisory boards to identify research priorities, and creating a Resource Library and Training Portal for stakeholders. The Core also collaborated with other Cores to provide training, mentorship, and funding for community-engaged research. Despite these achievements, geographical and cultural diversity presented engagement challenges. Regular meetings between investigators and stakeholders ensured bidirectional communication and aligned goals. The Core transformed transactional engagement into meaningful collaboration, emphasizing the need for interdisciplinary teams who understand community needs. Future goals include training academic teams, clinical providers, and community members, empowering early-stage investigators to share findings with partners, leveraging health records for research, and developing strategies to protect investigators’ time.

## Introduction

**Figure 1.**
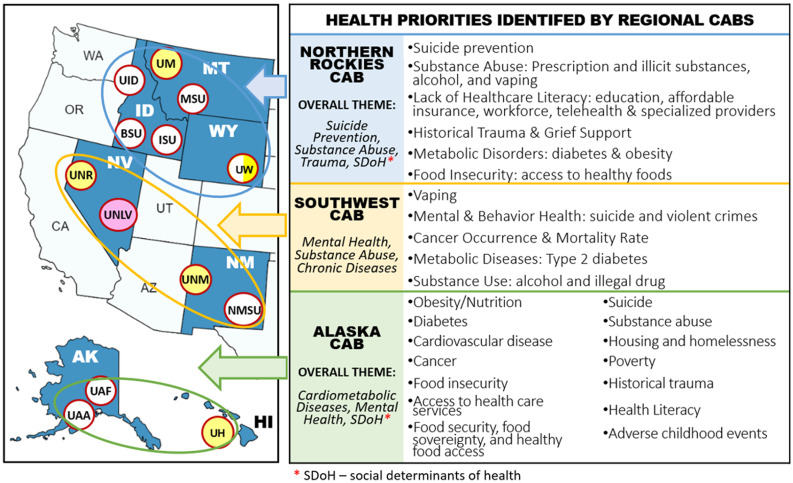
The IDeA states of the MW CTR-IN, University Cores, and Health Priorities (2013–2023). *This illustrates the Mountain West Clinical & Translational Research Infrastructure Network (MW CTR-IN, 1U54GM104944) between (2013 and 2023), which is made up of thirteen public institutions in seven Mountain West (MW) states, covering roughly one-third of the United States (US) and one-third of IDeA states. The common diseases and health challenges are identified by the three-regional Community Advisory Boards (CABs) specific to the individuals, families, and communities in their regions*.

The National Institute of Health (NIH) created the Institutional Development Award (IDeA) in 1993 to meet congressional mandates. The National Institute of General Medical Sciences oversees the initiative to expand NIH funding [1]. The IDeA program funds states with limited NIH funding to build a research infrastructure, facilitate basic, clinical, and translational research, and support the growth of early-stage investigators from diverse and underrepresented backgrounds. The IDeA initiative supported the Mountain West Clinical and Translational Research Infrastructure Network (MW CTR-IN, 1U54GM104944), which was made up of thirteen public institutions in seven Mountain West (MW) states, covering roughly one-third of the United States (US) and one-third of IDeA states (Fig 1).

The MW CTR-IN was awarded on September 15, 2013; its first cycle ended on June 30, 2018 [2]. It was dedicated to funding research studies on health inequities caused by disparities in economic and social resources, which prevent vulnerable and marginalized communities from participating in decision-making processes that affect their health and overall welfare [3]. The Network was extended for five years in August 2018 (2U54GM104944). In 2020, Hawaii received an IDeA Award (Pacific Innovations, Knowledge, and Opportunities), bringing the total number of institutions in six states down to 12.

The overall mission of the MW CTR-IN was to build and improve infrastructure to increase clinical and translational research (CTR) in the MW region. It started with three administrative leadership cores—Administrative, Tracking and Evaluation, and Clinical Pilot Projects—and two service cores for academic investigators—Professional Development and Biostatistics, Epidemiology, and Research Design. These cores were developed to interact and use the MW CTR-IN’s appropriate resources to foster collaborative research [2].

**Figure 2.**
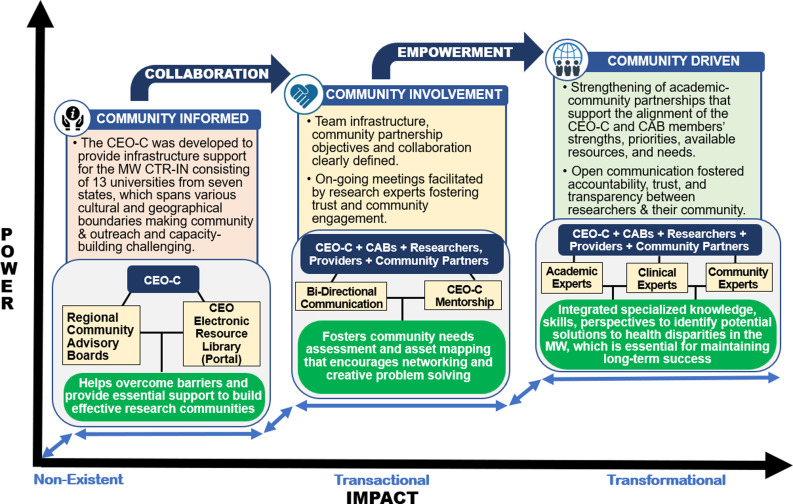
Transforming community engagement to advance health outcomes. *The community engagement & outreach core (CEO-C) built trust and shared decision-making, collaboration, and empowerment among academic-community partners to achieve transformational community engagement in the Mountain West*.

The Community Engagement and Outreach (CEO) Core was established in 2018 in response to the increasing emphasis on community engagement. The Core was designed to assist investigators in securing community-level support for successful research initiatives. The CEO Core established an infrastructure to address cultural differences, identify regional research priorities, and assist academic-community partners in project planning and implementation. Since the research priorities of the Network were centered around community health inequities, the CEO Core was tasked with building trust and shared decision-making, collaboration, and empowerment among academic-community partners to achieve transformational community engagement (Fig. 2) [4].

Although many of the six states connected to the MW CTR-IN include major towns with metropolitan centers, such as Albuquerque, Anchorage, Billings, Boise, Cheyenne, Las Vegas, and Reno, the region is primarily rural and frontier in terms of geographic area. Furthermore, much of the MW CTR-IN region is medically underserved, with a high proportion of racial and ethnic minority groups that confront considerable health inequities compared to other racial and ethnic minority groups and areas in the US[5]. Five MW CTR-IN states (Alaska, Idaho, Montana, New Mexico, and Wyoming) have rural populations above 34%, compared to 21% overall [6]. In New Mexico, 30 of 33 counties are health professional shortage areas, and 60% of the population lives in rural communities [7]. The largest American Indian/Alaska Native populations are in Alaska (15.5%), New Mexico (11.2%), and Montana (6.5%). About 20% of Hawaii’s population is Native Hawaiian Pacific Islander, and another 40% is Asian. With 50.2% and 30.3% Hispanic populations, New Mexico and Nevada are among the top five states with the largest Hispanic populations [8]. Due to expanding agriculture, MW CTR-IN states have more Hispanic migrant laborers [5].

Hence, the main challenge encountered by the CEO Core was the requirement to successfully engage our stakeholders and communities, considering their diverse makeup and wide geographical reach. Moreover, it was essential for the Core to educate other cores on the critical difference between community engagement and prior work conducted by investigators in communities or for communities rather than collaborating with communities as equal partners.

This case study outlines the primary initiatives and programs undertaken by the CEO Core since its establishment in 2018. We will share proven methodology and expertise that can assist other clinical and translational research centers and networks in developing community-based participatory research (CBPR) and community engagement initiatives. These initiatives aim to improve clinical and translational research and provide greater support for investigators, healthcare providers, and community members.

## An overview of the community engagement and outreach core of the MW CTR-IN

The NIH’s primary goal is to improve translational research capacity. In this context, the NIH recognizes the significance of community-engaged *research*[2]. This focus has grown in relevance due to the continued commitment to researching and reducing health inequities in developing chronic diseases. These challenges disproportionately affect vulnerable and marginalized communities in the US, including minority groups and people of color [3]. In 2018, Skinner and colleagues [9] advocated that funding agencies and academic institutions create policies, build infrastructure, and spend resources to improve investigators“ and universities” readiness to interact with the communities they serve and live in.

**Figure 3.**
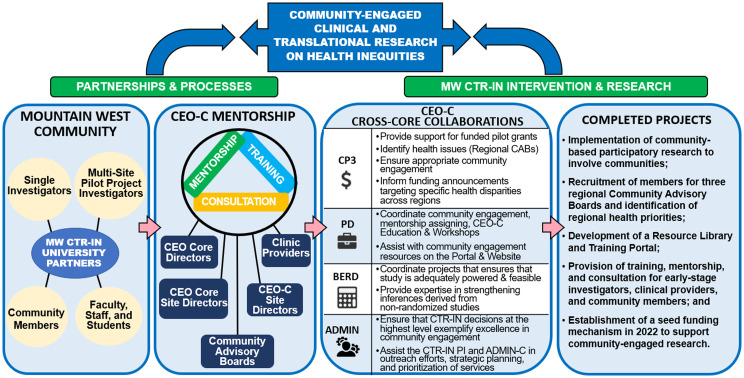
CEO core partnerships, mentorship processes, cross-core collaborations, & completed projects. *The community engagement & outreach core (CEO-C) provided its partnerships, mentorship processes, cross-core collaborations and completed projects to help ensure the success of academic-community partnerships of community-engaged CTR on health inequities in the Mountain West Clinical & Translational Research Infrastructure Network (MW CTR-IN, 1U54GM104944)*.

The MW CTR-IN’s CEO Core directly addresses the NIH’s goal of improving clinical and translational research while meeting the needs of the diverse communities in the MW region. When the CEO Core was developed and implemented, it became clear that a “one size fits all” approach to addressing health inequities across the MW would be ineffective and unacceptable. Thus, the CEO Core had to work with the communities and the other four MW CTR-IN Cores to improve the health of vulnerable and marginalized racial and minority ethnic groups who faced health inequities across the MW region by applying the community-based participatory research (CBPR) principles. In addition, the Core’s co-directors, associate directors, site directors, and community advisory boards (CAB) provided the mentorship, training, and consultation needed to ensure the success of academic-community partnerships (Fig. 3).

## Community engagement and outreach core initiatives (2018-2023)

The CEO Core’s primary purpose was to develop community partnerships to create dynamic local networks that optimize mentoring, resource sharing, and networking and propose community-owned solutions to health inequities and social justice issues [4,10]. This includes emphasizing empowerment and power-sharing mechanisms to address social inequities, leveraging community strengths and resources, encouraging collaborative learning and capacity development among all stakeholders, acknowledging the local significance of public health issues, and adopting ecological perspectives considering various health outcome factors. Intentional community-academic interactions can change perspectives, address social, cultural, and structural norms, and remove structural barriers, ensuring fair participation in all research procedures and recognizing each partner’s unique skills. We also wanted to prioritize initiatives that proactively provide capacity development opportunities like instruction, training, technical support, and logistical support like access to physical spaces and technology, which are essential for balanced partnerships.

**Figure 4.**
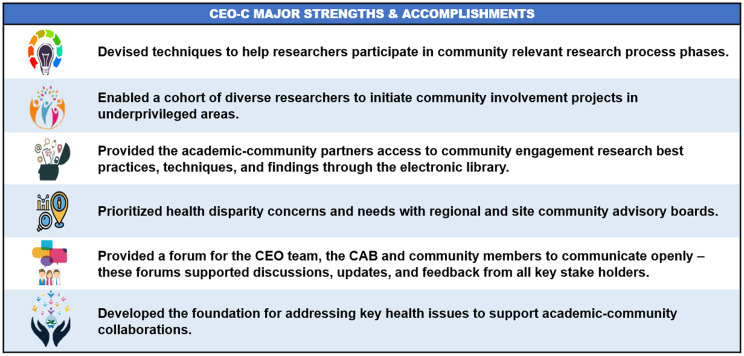
Major strengths & accomplishments of the community engagement & outreach core (2018–2023). *The concise overview of the community engagement & outreach core’s (CEO-C) major strengths and accomplishments during 2018–2023*.

Fig. 4 presents a concise overview of the CEO Core’s strengths and achievements during the last five years. Five initiatives led by the Core culminated in these accomplishments: 1) the creation of a Steering Committee tasked with forming interdisciplinary teams; 2) recruitment of members for three regional CABs and identification of regional health priorities; 3) development of a Resource Library and Training Portal; 4) provision of training, mentorship, and consultation for early-stage investigators, healthcare providers, and community members in collaboration with the other four cores of the MW CTR-IN; and 5) establishment of a seed funding mechanism in 2022 to specifically support community-engaged research.

### Creation of a steering committee tasked with forming interdisciplinary teams

To enhance stakeholder engagement, the CEO Core recognized the need to establish an interdisciplinary team that included investigators from diverse disciplines, project management professionals, and design experts. A Steering Committee for the CEO Core was established to independently address forming collaborative teams across various sites, states, and regions, separate from the Steering Committee of the MW CTR-IN. The committee was created to facilitate improvements in the research process and outcomes by enhancing research relevance, effectiveness, and sustainability through innovative stakeholder engagement strategies. It also led the CEO Core initiatives described in this case study. The committee included CEO co-directors (LSE, RS), who had expertise in community engagement, health disparities, public health priorities, and initiatives in the MW region, site directors for each university partner, a software engineer and informatics specialist, a project coordinator & graphical design expert (JGL), and the director of the Osher Lifelong Learning Institute in Nevada, who served as the community representative (RL). The CEO Core’s Steering Committee had monthly virtual meetings mostly focused on reporting back on the progress of the participating institutions and identifying initiatives and priorities of the Core.

The initial project of the Steering Committee of the CEO Core was to identify and pull together an interdisciplinary team of investigators from diverse fields and disciplines across the Network. A significant challenge addressed was the cultural shift among various departments and disciplines, alongside the establishment of a collaborative interdisciplinary team to support all institutional and community partners across the MW CTR-IN. Professionals educated and socialized within their specific fields found it difficult to shift their focus to coordinated interdisciplinary practice. They exhibited greater comfort within their specific disciplines, where they shared a common theoretical understanding, similar problem-solving approaches, and an established vocabulary.

The Steering Committee facilitated interdisciplinary collaboration by offering training materials via the MW CTR-IN web portal, including extensive resources on CBPR, community-engaged research, and community engagement. A directory of investigators who consented to collaborate on MW CTR-IN initiatives was compiled and uploaded to the Network’s web portal. This directory was updated monthly. The Network’s interdisciplinary team included physicians, nurse practitioners, clinicians, counselors/educators, social workers, pharmacists, physical therapists, engineers, computer scientists, sociologists, epidemiologists, and basic and life scientists while continuing efforts to recruit investigators from various fields and disciplines.

The CEO Core also collaborated with the other four Cores that composed the MW CTR-IN to plan, organize, and implement its annual conference. The annual conferences enabled face-to-face meetings among the investigators and solidified collaborative work. As new early-stage investigators were funded, some of the awardees also brought in additional senior investigators who joined the collaborative work of the Network. Existing investigators involved in the Network also referred colleagues to support interdisciplinary work within the Network.

### Establishment of three regional community advisory boards and identification of regional health priorities

The CEO Core aims to facilitate connections among investigators, healthcare providers, and community stakeholders. Thus, upon its inception, the Core sought methods to identify community stakeholders for active involvement. We were aware of the significant variations in race and culture among the MW CTR-IN states. To accommodate the extensive geographical area and diverse interests in different areas, we established three regional CABs. The initial CAB concentrated on Alaska/Hawaii, the second on the Rocky Mountain states (Montana, Idaho, and Wyoming), and the third on the southwestern states (New Mexico and Nevada). Approximately 20 community stakeholders volunteered to cooperate with the Core across the three CABs.

The regional CABs were utilized to identify regional-specific health priorities and distinct health needs shaped by various factors, including racial and ethnic minority group characteristics of the region, built environments, and social determinants of health [11]. Comprehending community context is fundamental to the evidence-informed approach in public health practice, wherein research and practical evidence are synthesized in decision-making. Regional CABs also allowed us to focus more on regional-specific health priorities and the uniqueness of their racial and ethnic minority groups and communities. Fig. 1 illustrates common diseases and health challenges identified by the three regional CABs specific to their regions’ individuals, families, and communities [12,13].

Community Advisory Boards collaborated with academic investigators, healthcare providers, and community members to facilitate their concentration on health issues unique to their respective regions. For instance, the areas that recognized mental health inequities in their communities were urged to tackle the particular health promotion requirements of the people they served based on the assumption that one environmental setting may not produce identical outcomes in another. Some CAB members also served as research advisors, team members, and participants in MW CTR-IN research initiatives. The CABs also helped identify and pair community partners and investigators who shared a commitment to reducing disease burdens for racial and ethnic minorities and promoting health equity [11,14,15].

The CEO Core’s Steering Committee organized quarterly virtual meetings to facilitate two-way communication among regional CABs. Furthermore, urgent meetings were scheduled as necessary to address critical issues. The Core conducted biennial virtual meetings with all regional CAB members to review site-specific accomplishments, challenges, and strategies for addressing them. The CEO Core website, accessible through the MW CTR-IN Portal, provides all uploaded meeting minutes for members who could not attend the virtual meetings. Commencing in 2016, yearly site visits were arranged to each of the 13 universities to interact with early-stage investigators, healthcare providers, and community members to rebuild relationships that the impersonality of virtual meetings had compromised. The primary investigator of MW CTR-IN, together with other members of the Network staff and leaders, took part in the site visits.

Finally, all CAB members were invited to attend the annual MW CTR-IN conference. During these meetings, the CABs provided vital insight into core goals, strategies, marketing tactics, and research distribution. Annual gatherings provided ideas and relationships for reducing health inequities among racial and ethnic minority groups. Community stakeholders were asked to give presentations and open forums to discuss issues with academic and community partners. These sessions addressed the demands of the MW region and issues raised by community members. Both formal and interactive talks centered on how research impacted daily living. Lastly, CAB members were invited to sessions where grantees from their regions presented their project findings at conferences. They actively engaged and provided feedback after the presentations to strengthen the project’s quality and rigor and guide pilot grantees in preparing their manuscripts for dissemination.

### Creation of a resource library and training portal

Expansion of the MW CTR-IN Resource Library and Training Portal was another initiative of the CEO Core. An extensive collection of scholarly and media materials about CBPR, community-engaged research, community engagement, cultural relevance, ethics, justice and equity, chronic illness management, research involving human subjects, rural health, and vulnerable and marginalized communities may be found on this website. Training materials, documentaries, reports, and peer-reviewed research publications are examples of community participation in action. Talks about relevant and current issues were started during the CEO Core meetings. In the annual virtual meeting with regional CAB members, gaps in community involvement training were identified, prompting the arrangement of an open forum to discuss required additional resources. Improvements have been made to the Training Portal and Resource Library as a result of these discussions.

In addition to the Resource Library and Training Portal, each CEO Core site director sponsored one yearly training workshop. These monthly trainings were primarily related to health inequities research in the MW region and consisted of recorded webinars and PowerPoint presentations shared with investigators to support community engagement. Examples included a video Webinar on “Best Practices in CBPR and CAB Development” and a toolkit for “Dissemination of Rural Health Research.”

### Training, mentorship, consultation, and funding for community-engaged research

The CEO Core is essential for early-stage investigators, healthcare providers, and community members (e.g., primary healthcare centers) who require community engagement assistance. Investigators and Core site directors consult before engaging in a research project to ensure they have the necessary support and that their application includes a community engagement strategy. Consultants benefit early-stage investigators and new faculty lacking community engagement experience. The Core works with a diverse range of investigators. Identifying campus and community locations to recruit Latinx students aided one project. A community partner who assisted veterans with posttraumatic stress disorders was connected to an early-stage investigator who studied veterans to support another project. In another case, the Core linked an early-stage investigator with a small group of community leaders to organize a workshop to initiate a community dialogue about suicide rates and launch a community development initiative to address the issue.

Early-stage investigators and pilot awardees are also instructed to consult site directors via the portal before submitting MW CTR-IN applications. This practice is critical because it enables early-stage investigators to understand their community involvement responsibilities. The CEO Core site directors guide investigators regarding their research objectives and the target racial and ethnic minority groups, aiding them in overcoming obstacles such as language barriers and cultural differences. They also recommend community health worker networks, community health agencies, and research assistants fluent in Spanish and native languages to bolster and broaden their infrastructure for community-based research. Investigators were also required to regularly submit a summary of their research progress through the portal. This summary included the study site, health inequities topic, and the principal investigator’s bio.

The process of seeking advice for mentoring related to pilot projects or research studies starts with the mentee submitting a request in the portal to meet with a mentor. A member of the CEO Core is expected to respond within 24 to 48 hours to follow up on the assistance required by the individuals. When the CEO Core could not address a mentee question, suitable referrals were provided. The Core designee recorded the details of the consultation and the resolution of any identified issues. To assess satisfaction with the consultation, stakeholders who submitted a query received an email to evaluate their satisfaction regarding the resolution of their problem or the response to their query.

Finally, early-stage investigators and all pilot grant awardees were instructed to meet with their institution’s site director at the start of their MW CTR-IN project. This kick-off meeting enabled the assessment and verification of assistance needed from the CEO Core for each project. Site directors facilitated site recruitment, leading to expedited project completion. For example, in 2022, an early-stage investigator at one of our sites received pilot funding to conduct an ethnic study to build strength for mental health in racial and ethnic minorities. This pilot grantee asked the site director for assistance contacting Alaska Natives, Filipinos, Samoans, and African Americans in their region to participate in the study. They received more than 2,000 responses from 40-45 states, exceeding their target of 650. A meeting schedule for the one-year funding period was also established during the kick-off meeting. Site directors engage with pilot grant awardees throughout the study period to offer supplementary mentorship and conduct periodic check-ins.

After the funding period, site directors helped pilot grantees interpret and contextualize their findings and advised them on future research. Early-stage investigators received ongoing support and guidance from site directors to improve research outcomes and community well-being. They encouraged them to present and publish their findings at conferences and share them with their communities via extensive networks and multiple platforms. For example, one site director helped a pilot grantee choose meetings and journals to publish a preliminary study on immigrant mental health interventions. This guidance can boost research visibility and significance. Finally, site directors also assisted pilot grantees with developing strategies for culturally sensitive health interventions.

The CEO Core also collaborated with the Professional Development Core to organize six annual Grant Writing Workshops available to all early-stage and mid-career investigators. A CBPR workshop was also held in 2022 to assist early-stage investigators interested in CBPR methodologies and community-engaged research [16]. The workshop was designed for prospective applicants for the Community Engagement Research Pilot (CERP) award and led by prominent CBPR investigators alongside their community partners.

### Establishing seed funds for community engagement research projects

The MW CTR-IN funded CERP projects in the fourth year of the second funding period. These grants supported community engagement and regional CAB issue-focused research. The initiative selected clinical and basic science pilot studies using the same competitive process. The process included a request for proposals, informative sessions, an initial assessment, letters of intent, and finding a community partner for the pilot project. The selection process prioritized applications focused on fair and equal partnerships and community-based health research [4]. These efforts included community evaluation, health promotion and education, intervention testing, and program evaluation. Early-stage investigators had to include a community partner in their proposals to promote equitable power distribution among partners [10]. We used a cyclical methodological model with community leadership, input, and feedback to set mutually beneficial goals [10,17]. This collaborative approach engaged all community stakeholders equally in the research project and designated budgetary funds to support their organizations.

The CEO Core leaders and collaborators, including previous pilot grantees, healthcare providers, and community members with expertise related to a CERP application, were invited to review the applications. The reviewers for the CERP applications must answer questions like: 1) Can the pilot study and future funding proposals improve health disparity among racial and ethnic minority groups? 2) Is the pilot research culturally appropriate? 3) Do community partners participate equally in the pilot research project?

Involving previous pilot grant awardees, healthcare providers, and community members in grant reviews improves applications, especially those focusing on community engagement and health inequities. Assigned reviewers are expected to utilize their expertise to assess projects. In a review, an assigned reviewer may emphasize the importance of community feedback in the research design of a proposed intervention to address a health disparity. This ensures alignment of the project with the community’s needs and priorities. The review process underscores the importance of the CEO Core in ensuring that projects have a community engagement strategy and the requisite resources to engage with the community and achieve their objectives.

## Conclusion

The MW CTR-IN has achieved significant progress over the past five years. These encompass the development of strong interdisciplinary teams across the Network, enhanced networking and collaboration between academic institutions and the community, improved communication among early-stage investigators via training, mentorship, and consultation, the implementation of strategies to promote community partnerships, increased productivity among sites, early-stage investigators examining health inequities in vulnerable and marginalized communities, and funding for pilot studies centered on community engagement. The Core’s achievements are crucial because community involvement sustains health and reduces health inequities. Community engagement ensures responsible and transparent practices in clinical and translational research and public health initiatives [18]. The MW CTR-IN CEO achieved the outcomes detailed in the case study while addressing the distinctive challenges of advancing research on racial and ethnic minority groups in underserved communities and fostering meaningful community engagement through collaboration between Community Advisory Boards (CABs) and academic and community partners. This paper’s reflections may help other organizations reduce racial and ethnic minority health inequities through clinical and translational research.

Although we have achieved significant milestones, the MW CTR-IN, specifically the CEO Core, requires further enhancement of our clinical and translational research initiatives. The objective is to continue applying CBPR and community engagement principles that align with the MW region’s unique geography and diverse demographics. Investigators, healthcare providers, community members, and other stakeholders may develop research questions during study planning to determine important outcomes. Study materials and protocols may be created or modified during the implementation phase, and recruitment or data collection may be conducted. A dissemination plan may be established, and appropriate partners may be selected to disseminate results. Our objective is to improve initiatives for identifying, recruiting, and retaining study participants who represent the diverse racial and ethnic minority groups of the MW region. We will also employ patient-reported outcomes, as those affected or at risk of a condition offer the most pertinent information regarding the outcomes of interest following the standards established by the Patient-Centered Outcomes Research Initiative [19].

If we are successful in a third renewal, the CEO Core will continue to support research on health inequities. The MW CTR-IN will grow its training programs to include leadership and research methodology workshops for investigators, healthcare providers, and community members (i.e., community health workers). We will also expand the Resource Library and Training Portal, hold monthly training sessions on best practices for community interaction, and make the training available beyond our Network. We will also expand our current infrastructure to establish a formal Practice-Based Research Network where healthcare providers’ knowledge and expertise are used to solve clinical practice-related research problems [20]. By connecting clinicians in healthcare practice settings and our community partners through CAB, we aim to identify key study areas and provide results that are broadly relevant, easily applicable, and easily implemented in everyday healthcare.
